# How accurate are we at assessing others’ well-being? The example of welfare assessment in horses

**DOI:** 10.3389/fpsyg.2014.00021

**Published:** 2014-01-24

**Authors:** Clémence Lesimple, Martine Hausberger

**Affiliations:** Laboratoire d’Éthologie Animale et Humaine EthoS, UMR CNRS 6552, Université de Rennes 1Rennes Cedex, France

**Keywords:** well-being recognition, bad-being decoding, questionnaire reliability, horse, abnormal behavior

## Abstract

Healthcare practitioners such as physicians or nurses often underestimate patients’ well-being impairment (e.g., pain, anxiety) which may lead to undesirable consequences on treatment decisions. Lack of recognition/identification of signals and over-exposure are two reasons invoked, but a combination of factors may be involved. Studying human decoding of animals’ expressions of emotions showed that “identification” to the subject was necessary to decode the other’s internal state. In the present study we wanted to compare caretakers’ reports on the prevalence of stereotypic or abnormal repetitive behaviors, to ethological observations performed by an experienced observer on the same horses in order to test the impact of these different factors. On the first hand, a questionnaire was given hand to hand to the caretakers. On the other hand, the experienced observer spent 18 h observing the horses in each stable. Here we show that caretakers strongly underestimate horses’ expressions of well-being impairment. The caretakers who had a strong concern about their horses’ well-being were also those who reported the more accurately SB/ARB’s prevalence, showing that “identification” to the subject is a primary factor of bad-being signal’s detection. Over-exposure also appeared to be involved as no SB/ARB was reported in stables where most of the horses were performing these abnormal behaviors. Being surrounded by a large population of individuals expressing clear signals of bad-being may change professionals’ perceptions of what are behaviors or expressions of well being. These findings are of primary importance as (1) they illustrate the interest of using human-animal relationships to evaluate humans’ abilities to decode others’ states; (2) they put limitations on questionnaire-based studies of welfare.

## INTRODUCTION

The concept of well-being is described according to two main approaches: the hedonic one comparing the amount of positive versus negative effects; the eudaimonic approach, where it is described as the satisfaction of the physiological and physical needs of an organism (Tomer, 2011 for a review). Identification of the other’s well-being is raising more and more interest throughout the scientific community. However, decoding and recognizing others’ pain or affected internal state (altered homeostasis) is very important in particular in a context of medical assistance or welfare evaluation while they are often poorly and/or under-evaluated (Ahlers et al., 2010; Topolovec-Vranic et al., 2010). Thus it has been repeatedly shown that healthcare practitioners, in particular nurses, tend to underestimate the severity of patients’ pain, and consequently of their welfare impairment (Marquié et al., 2003; Hirsh et al., 2011). Such miscalibration may affect treatment decisions and hence individuals’ quality of life (Prkachin et al., 2004). More surprising is probably the fact that nurses underestimate facial expressions of pain more than do people with less experience (Prkachin et al., 2004). Several factors can explain these findings: (1) over-exposure appears to reduce sensitivity to facial expressions of emotions (Prkachin, 2002): high intensity expressions may become the standard and thus lower expressions are downgraded; (2) demographic characteristics of patients influence the evaluation: (a) emotional facial expressions are better decoded by individuals of the same national, regional, or ethnic group (Thibault et al., 2006), (b) risk of suboptimal pain care increases for people from a social minority [gender, ethnic group, age (Auret and Schug, 2005; Kamath and O’Connor, 2011)]. This may be due to the difficulty to take the others’ perspective, hence accuracy is better when individuals have to identify the expressions of people who share their racial background (Elfenbeim and Ambady, 2002).

In an attempt to control ingroup/outgroup effects, Thibault et al. (2006) used pets (cats) as encoders to test emotion recognition by humans. Accuracy appeared to be low and was not influenced by experience (e.g., number of years of experience), liking or contact with cats, but was influenced by “identification” (“pets are like us”). Identification may have induced “decoders” to attempt to take the cat’s perspective and hence to be more motivated to engage in cognitively more demanding decoding strategies. Indeed, humans’ decoding of other species’ expressions of emotions, pain, or poor welfare constitutes an interesting conceptual framework to test factors influencing the assessment of others’ well-being. Thus, people tend to assess rabbits’ pain by looking at their faces while this species expresses pain mostly through body postures (Leach et al., 2011). However, to our knowledge, there was no attempt to compare professionals’ estimation and scientific objective evaluation of this welfare impairment.

The present study aimed to test the ability of experienced caretakers in assessing horses’ expression of poor welfare, hence visible abnormal repetitive behaviors (ARB) and stereotypic behaviors (SB), which are recognized as signals of welfare impairment (Mason, 1991). SB/ARB have been shown to relate to poor welfare conditions (Mason, 1991) and to occur regularly in the restricted social/feeding/spatial conditions under which horses are kept (Mills, 2005). Discrepancies can be observed in the literature concerning their prevalence in adult working horses (from 1 to 96%; Parker et al., 2008; Hausberger et al., 2009). A thorough examination of these studies reveals that two types of approaches: surveys (questionnaires to caretakers) and direct observations by researchers led to different results, with prevalence varying from 1 to 10% in the first case and from 22 to 97% in the second. We hypothesized here that horse caretakers do, like human healthcare practitioners (nurses and physicians, Prkachin et al., 2004; Lidén et al., 2012), underestimate horses’ expressions of discomfort, even though these expressions may be visible. We propose that two types of factors are involved: (1) lack of signals’ identification or recognition, (2) over-exposure to individuals with bad-being expression leading to lowered sensitivity.

## MATERIALS AND METHODS

### ANIMALS

This study concerned 373 horses of various sex (212 geldings, 159 mares, and 2 stallions), ages [3–34 years (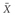 = 12.5)] and breeds (*n* = 24, mostly unregistered horses: 35%, French saddlebred: 26%, French ponies: 15.5%, and smaller proportions of Anglo-Arabians, Connemara, Mérens, and New-Forest) in 26 riding schools all over France (14.3 ± 1.5 horses per school). The horses were under the management of the riding schools, housed mostly in individual boxes/stalls (93.8%) or in individual (4.3%) or group (1.9%) paddocks. Most of them were fed pellets (93%), one to three times a day, while some (*n* = 13.7%) had no concentrates. Most (89%) of them also had hay distributed in one to five meals. A few riding schools could not provide hay (*n* = 55 horses). All horses had water *ad libitum*.

Horses worked 4–20 h a week in riding lessons involving mostly children and teenagers, with one closing day.

### TERMINOLOGY

Stereotypic behaviors are defined as unvarying, repetitive and apparently functionless behavioral sequences (Mason, 1991). To facilitate the reading of the following manuscript, we call here “stereotypic behaviors” (SB) the sequences well-known in the horse industry (e.g., weaving, cribbing) and “ARB” the sequences less (not) described or recognized (Mills et al., 2002; Mills, 2005). We describe below the five SB and the nine ARB observed or reported in this study.

#### Stereotypic behaviors

– Weaving: obvious lateral movement of head, neck, forequarters, and sometimes hindquarters,
– Cribbing/windsucking: the horse grasps a fixed object with its incisors, pulls backward and draws air into its esophagus,
– Head tossing/nodding: vertical movements of head and neck,
– Striking with forelimb: the horse hits the door or wall with one of its forelegs,
– Box walking: repetitive tracing a route within the stable.

#### Abnormal repetitive behaviors

– Compulsive licking: repetitive licking of the same object in its environment (except the trough),
– Compulsive biting: repetitive biting of the same object in its environment (except the trough),
– Head movements (other than head tossing/nodding): repeated movement of the head,
– Aimless threats: the horse express threat sequences (kicking, biting) alone in its box,
– Mouth open: the horse keeps its mouth open with a lateral movement of its neck,
– Teeth rubbing: rubbing teeth on the upper part of the door,
– Teeth chattering: mouth movement with teeth chattering,
– Lips movements: clapping of lips,
– Tongue movements: movements of tongue, inside, or outside the mouth

### QUESTIONNAIRE

In each riding school (*n* = 26), the person who was the most familiar to the horses (horse’s caretaker involved both in daily and health care, who, in some cases, was also the owner) was asked to answer a questionnaire (**Table 1**) about whether the horses in their care presented any chronic disorders, including the presence and type of SB and ARB. The questionnaire was created by the authors and intentionally not specific to the evaluation of SB/ARB, to evaluate whether, in a global context including all possible chronic disorders, SB/ARB could be identified. Before giving the questionnaire, the experimenter gave the definition of SB and ARB, and the following instructions were given to all respondents: tick the right boxes when possible, and describe the type of SB or ARB reported if necessary. Respondents were encouraged to report any supplementary comment they considered useful, concerning the possible causes of chronic disorders. However, all of them only ticked boxes.

**Table 1:** Questionnaire given to the horses’ caretakers.

Name of the horse	Nothing to report	Lamensess	Allergy	Cough	Ocular discharge	Sensitive to colic	Back pain	Stereotypy		Other chronic disorders
								Yes/No	Type of stereotypy
										

*The questionnaire included several possible chronic disorders and was voluntarily not focused on SB/ARB, in order to avoid potential biases. The questionnaire was completed for every horse in every school. Respondents were asked to tick the right boxes, and could add remarks in the column “other chronic disorders” or on the verso of the page.*

Questionnaires were given hand to hand, and they were filled in for each horse in every riding school. Thus we had for each of the 373 horses observed the evaluation of presence/absence of SB/ARB and their type or description.

### OBSERVATIONS

The aim was to assess differences between questionnaire surveys and scientific observations concerning the prevalence of stereotypic behaviors, the reasons of their emergence will be treated in a subsequent manuscript (Lesimple et al. in preparation). The observer was a fully trained ethologist who had performed a PhD on horse’s welfare and hence had trained with two experienced ethologists (M. Hausberger and C. Fureix) who had already defined and described ARB (e.g., Hausberger et al., 2007, 2009; Benhajali et al., 2010; Fureix et al., 2011) in accordance with earlier studies from other groups as well (e.g., Mills, 2005). The work started once the three observers had been in total accordance on the definitions of ARB/STB. Since ethology is fundamentally a science of observation, the quality of observational skills was the first criterion of training. To our knowledge, there is no demonstrated gender effect on the identification of such obvious behaviors as are ARB/STB. Observational training is not given to professional horse caretakers, at least in France. The experimenter stood in the stable for 6 h for three consecutive days (from 9 to 12 h am and from 14 to 17 h pm), at a place where she could see all the horses (individual boxes were placed in two rows separated by a corridor). In all riding schools, horses performed ARB/SB when they had the head at the box door. Thus, she was able to observe each horse in its box, 6 h a day (i.e., 18 h observation for each horse). As the aim was to evaluate the prevalence of abnormal behaviors, she recorded using the *ad libitum* sampling method each time a stereotypic behavior appeared and for which horse. Thus we had an exhaustive list of SB / ARB for the entire population.

### DATA ANALYSES

As data were not normally distributed, we used non-parametric statistical tests. Spearman’s correlation tests and Chi Square tests were used to detect the effects of age, gender, and site on the prevalence of stereotypies observed by the experimenter. Chi square tests and Cohen’s kappa agreement coefficient were used to assess differences between the experimenter’s observations and questionnaire evaluations.

## RESULTS

**FIGURE 1:**
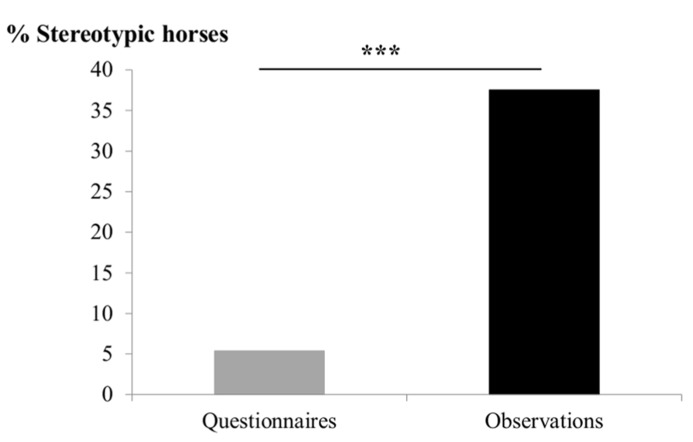
**Prevalence of horses presenting stereotypic behaviors according to questionnaire evaluation on the left and observations on the right.** The proportion of horses observed presenting SB/ARB is seven times that reported by questionnaires. Chi square test, ^***^p < 0.0001.

The observations revealed that 37% (*n* = 140) of the horses expressed at least once a stereotypic / ARB SB (in fact the minimum observed was five times in 3 h for a given horse; **Figure 1**). Horses presented 1 (*n* = 94), 2 (*n* = 28), or more (3–7, *n* = 18) types of SB/ARB. Twenty-six schools were involved, and prevalence varied greatly between schools (0–100% of the population, χ^2^ = 58.5, *p* < 0.001). In only two schools, no horse could be observed displaying any stereotypic behavior (0% of the population).

The caretakers’ evaluations showed a very large discrepancy with these findings: they indicated a prevalence of only 5% (*n* = 21) of horses (χ^2^ = 124.7, *p* < 0.001; **Figure 1**) and reports of SB/ARB came from only 13 schools. No horse was reported to present more than 1 SB. Agreement was poor between both evaluations (Cohen’s kappa = 0.14).

Interestingly, caretakers from schools where only a small proportion of horses were involved (0–2 horses) did not report any SB/ARB in the questionnaires (*n* = 6 schools).

Underestimation of these indicators of poor welfare therefore was high, indicating a potential lack of identification and hence motivation (or attention) to decode the behavior.

**FIGURE 2:**
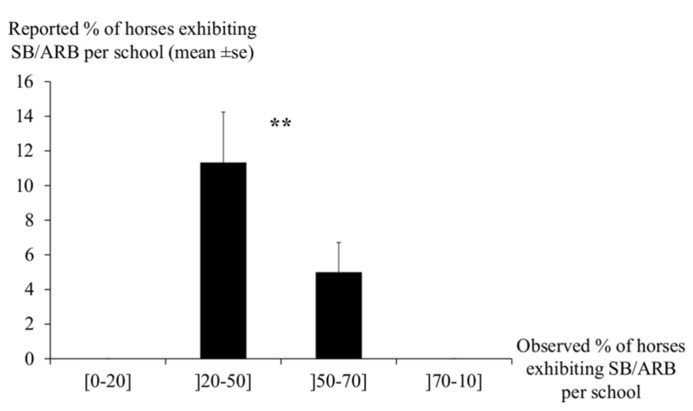
**Reported percentage of horses with stereotypic behaviors (questionnaires) according to the observed percentage of horses with stereotypic behaviors (observations) per school.** The error bars represent standard errors. Respondents evaluated the presence of stereotypic behaviors the best when 20–50% of horses were affected and the worst when 0–20 or 50–100% of horses were affected. Kruskall–Wallis nova, ^**^p = 0.02.

Over-exposure may also be involved as (1) the difference between the observed prevalence per school and the degree of discrepancy were correlated (Spearman correlation’s test, *r*_s_ = 0.9, *p* < 0.001): the higher the proportion of horses involved, the higher the discrepancy, (2) the lowest reports came from either stables with either the lowest (0–20%, *n* = 6 schools) or the highest (>50%, *n* = 7) prevalence of SB/ARB while more realistic reports were given when 20–50% of the horses were involved (*n* = 13; Kruskall–Wallis ANOVA, *H*(2, *n* = 26) = 8.2, *p* = 0.02; **Figure 2**).

**FIGURE 3:**
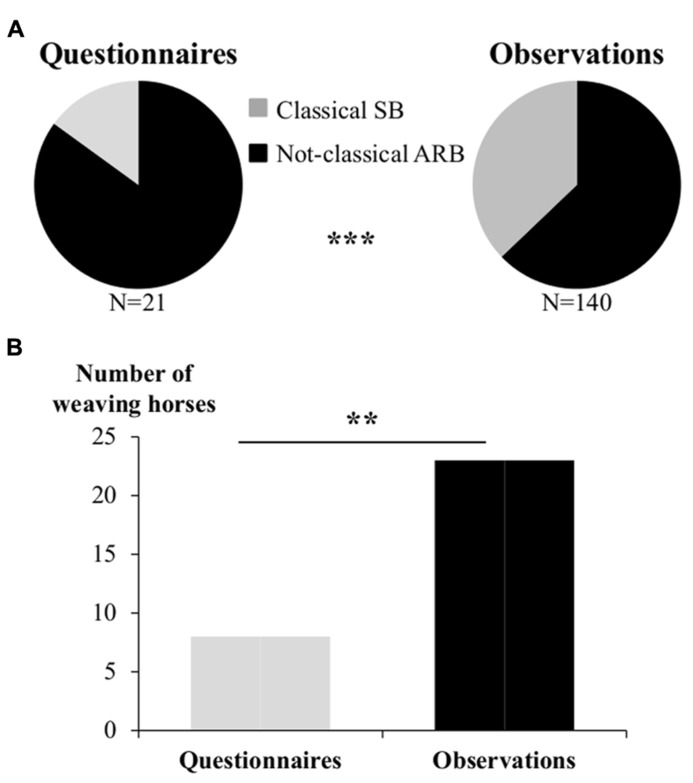
**(A)** Observed (on the left) and reported (on the right) relative proportions of SB and ARB in the test population. Chi square test, ^*^^*^^*^p < 0.001. **(B)** Example of one major SB: weaving. Note the important under-evaluation of weaving prevalence by questionnaires. Chi square test, ^*^^*^p < 0.01.

The third factor tested was potential difficulties to assess subtle differences (which may also be due to lack of identification). Indeed, “minor” ARB were still less reported by caretakers than “major” SB (observed: 63% SB, 37% ARB; reported: 85% SB, 15% ARB, χ^2^ = 12.6, *p* < 0.001; **Figure 3A**).

Therefore, more subtle/less known expressions were less detected, nevertheless, even when only “major” SB were considered, a strong discrepancy was still observed between observations and questionnaires. Thus while 23% of the 373 horses were observed weaving, only 8% were identified by the caretakers (χ^2^ = 7.6, *p* < 0.001; **Figure 3B**).

## DISCUSSION

Horse caretakers, like human health care practitioners (Prkachin et al., 2004; Lidén et al., 2012), clearly underestimate expression of bad-being in horses, despite the fact that SB are clearly visible and known in professional circles. The factors involved seem to be similar to those implied in the underestimation of pain and anxiety of patients by nurses and physicians (Prkachin et al., 2004; Lidén et al., 2012): potential lack of identification and over-exposure. Thibault et al. (2006) argued that “identification” to the subject was a primary factor for decoding its emotions, by enhancing the motivation to make a cognitive effort, while experience (in this case with pets) did not increase accuracy. Interestingly, the prevalence of accidents of professionals with horses has been shown to depend more upon exposure than experience (Jaeggin et al., 2005): accuracy to detect cues of imminent aggression does not seem to increase with time in contact with the animals. In the present case, two of the three schools where professionals expressed a strong concern for the horses’ welfare had a low prevalence of SB (0–10%) and a corresponding low report (0%), while the third had a higher prevalence (30%) but the report was quite accurate (30%). Obviously, as suggested by Thibault et al. (2006), individuals who identify themselves more to the subject may put more efforts in detecting signs of poor well-being (and prevent it from occurring). The effort required is stronger in the case of more subtle/less known signals, which explains that “minor” forms of ARB were still less detected.

Over-exposure appeared also here as a major factor and the findings support the idea that the abnormal behaviors observed may somehow become the standard: the riding schools where more than 70% of the horses expressed SB were also those where discrepancies between observations and reports were the highest. A previous study indicated that the evaluation of the potential negative impact of stereotypic behaviors by owners of horses presenting these behaviors was lower than by owners of horses that did not display such behaviors (McBride and Long, 2001). Being surrounded by a large population of suffering individuals may change human healthcare workers’ as well as horses caretakers’ perceptions of what are “normal” behaviors or expressions. Thus the presence and intensity of patients’ anxiety is strongly underestimated by both experienced nurses and physicians during surgery (Lidén et al., 2012). Except from professionals specifically trained to detect expressions of bad-being (e.g., depression) such as psychologists for example, people exposed to a majority of individuals whose welfare is impaired might be less sensitive to abnormal behaviors or bad-being signals (Prkachin et al., 2004). In fact, their perception of what is “normal” might be downgraded and integrate more or less subtle “bad-being” signals. The fact that relative exposure to human faces displayed on screens and expressing various levels of pain influences the observers’ sensitivity (Prkachin et al., 2004) supports an adaptation-level effect (Helson, 1964; Rollman, 1979). The findings that evaluations of prevalence of horses’ SB were strongly influenced by the real proportion of SB horses strongly support the idea that this effect is involved here too, because professional caretakers are not specifically trained to detect such signals.

An alternative hypothesis to explain the under-evaluation of SB/ARB could be that people were reluctant to report behavioral problems in their horses. As SB/ARB are known to occur under sub-optimal conditions, questionnaire respondents may be tempted to give answers that will reflect positively their institution (Meagher, 2009). However, several elements suggest that this is not the main explanation: (1) respondents were generally not the owners of the structure and thus not as much involved in how the school was perceived, (2) they saw the experimenter during the observations and knew that she worked, amongst others, on the SB/ARB. It is thus unlikely that they tried to hide the presence of horses’ abnormal behaviors.

These findings are of major importance for three main reasons: (1) they suggest that using human evaluations of domestic animals’ expressions may be a precious conceptual framework to evaluate humans’ abilities to decode others’ internal states; (2) they reveal major limitations to studies of humans’ or animals’ well-being entirely based on questionnaire surveys; (3) underestimations of well-being impairment by caretakers and health workers are very worrying as they may lead to inappropriate or insufficient treatment decisions (Prkachin et al., 2004). Furthermore, the idea that over-exposure, via the adaptation-level effect, may lead to lower cognitive efforts due to a lack of identification and thus to a decreased perception of subtle signals, is worrying. Indeed, whether in the context of human medical care or animal welfare, the underestimation of bad-being signals can lead to inappropriate treatment or management with possible serious consequences.

However, these findings also suggest solutions: training professionals to observe subtle signals, giving them regular access to “normal” subjects and motivating them by developing their empathy (giving them the subject’s perspective, enhancing identification) could have a major positive impact. Thus the problems of identification of bad-being signals due to the adaptation process might be solved or at least substantially decreased, and the overall large problem that such underestimations induce diminished [see also programs of observational training for neonates’ caretakers (Als et al., 1977)].

To the extent that this study is mostly based on the observation of one experienced ethologist, further research could be conducted to compare the evaluation of SB/ARB between several experienced ethologists. Moreover, it would be interesting to compare the detection of such behaviors between experienced ethologists and horse professionals (e.g., caretaker) trained to observation.

## AUTHOR CONTRIBUTIONS

Clémence Lesimple and Martine Hausberger designed the experiments, Clémence Lesimple performed the experiments, Clémence Lesimple analysed the data, Clémence Lesimple and Martine Hausberger wrote the paper.

## Conflict of Interest Statement

The authors declare that the research was conducted in the absence of any commercial or financial relationships that could be construed as a potential conflict of interest.
